# 3,3′-Diindolylmethane modulates aryl hydrocarbon receptor of esophageal squamous cell carcinoma to reverse epithelial-mesenchymal transition through repressing RhoA/ROCK1-mediated COX2/PGE_2_ pathway

**DOI:** 10.1186/s13046-020-01618-7

**Published:** 2020-06-16

**Authors:** Peiyao Zhu, Huayun Yu, Kun Zhou, Yu Bai, Ruiqun Qi, Shuguang Zhang

**Affiliations:** 1 Department of Thoracic Surgery, The First Hospital of China Medical University, No.155 North Nanjing Street, Shenyang, 110001 China; 2 Department of Gynecology and Obstetrics, Clinical Medical School, Shandong University, 44# Wenhua Xi Road, Jinan, 250012 China; 3 Department of Dermatology, The First Hospital of China Medical University, No.155 North Nanjing Street, Shenyang, 110001 China

**Keywords:** ESCC, AHR, DIM, EMT, RhoA, ROCK1, COX2, PGE_2_

## Abstract

**Background:**

Esophageal squamous cell carcinoma (ESCC) is one of the most aggressive tumors in the world. Aryl hydrocarbon receptor (AHR) has been reported to promote tumor metastasis and epithelial-mesenchymal transition (EMT) is a vital process of conferring cancer cells capabilities of migration and invasion. However, the mechanism by which modulation of AHR can inhibit tumor metastasis remains unknown. Thus, we aim to investigate the underlying mechanism regarding reversing EMT process of ESCC through modulation of AHR.

**Methods:**

We used AHR selective modulator 3,3′-diindolylmethane (DIM) to treat ESCC cell lines TE1 and KYSE150 so as to examine alterations of migration and invasion by wound healing and Transwell assay. Western blotting (WB) and qPCR were performed to detect relative genes and proteins changes regarding EMT process. Cell transfection was utilized for confirming pathways involved in DIM-induced reversal of EMT and in vivo assay was conducted for verification of the underlying mechanism. Co-IP assay was conducted for detecting protein-protein interactions.

**Results:**

AHR was overexpressed in ESCC and modulation of AHR by DIM could inhibit migration and invasion as well as downregulate mesenchymal cell markers β-Catenin, Vimentin and Slug and upregulate epithelial cell marker Claudin-1. Meanwhile, synergically overexpression of AHR, RhoA and ROCK1 correlated with poor clinical outcomes. DIM could inhibit COX2/PGE_2_ pathway by targeting AHR, and COX2 selective inhibitor Celecoxib could suppress EMT and metastasis. Results of PGE_2_ treatment were opposite to that of Celecoxib. Meanwhile, blockade of RhoA/ROCK1 pathway also exerted prohibitive effects on EMT and metastasis. WB results showed COX2/PGE_2_ pathway could be regulated by RhoA/ROCK1 pathway and DIM could inhibit RhoA/ROCK1 pathway through modulation of AHR. In vivo assay verified the results in vitro. Co-IP results showed DIM could modulate AHR to reverse EMT directly through inhibition of interaction between AHR and EGFR (epidermal growth factor receptor) so as to block RhoA/ROCK1-mediated COX2/PGE_2_ pathway which was connected by NF-κB.

**Conclusions:**

In brief, modulation of AHR by DIM can reverse EMT process and inhibit metastasis of ESCC through repressing RhoA/ROCK1-mediated COX2/PGE_2_ pathway.

## Background

Esophageal carcinoma remains one of the most aggressive malignant tumors and according to recent reports, it ranks seventh regarding incidence of cancers and sixth in mortality worldwide [1]. Esophageal squamous cell carcinoma (ESCC) is the main histologic subtype within the scope of lower income countries, especially in parts of Asia and Africa [2]. Due to its rapid progression towards advanced stages with strengthened capability of metastasis, early diagnosis and early treatment make sense. Despite of traditional therapies, targeted therapy emerges a more promising method for ESCC [3]. Therefore, identification of novel genes that may function as biomarkers and developing effective agents targeting these genes are of significance to improve quality of patients with ESCC.

3,3′-Diindolylmethane (DIM) is an active metabolite of indole-3-carbinol (I3C) found in cruciferous family. Evidences show that I3C can exert its anti-tumor properties through regulating cell growth, cell cycle and division, apoptosis and metastasis mainly through aryl hydrocarbon receptor (AHR) [4]. Meanwhile, AHR is a ligand-dependent transcription factor and has been found abnormally elevated in some tumors including breast cancer, non-small cell lung cancer, colon cancer and ovarian cancer [5–8]. Once binding to a proper ligand, AHR will translocate into the nucleus forming complex with AHR nuclear translocator (ARNT) for locating on the xenobiotic response elements (XREs) region to regulate transcription of downstream genes [9]. Our previous study have reported that both knockdown of AHR and modulation of AHR by DIM could inhibit ESCC growth, induce cell cycle arrest and promote apoptosis [10]. Emerging evidence shows that AHR plays a crucial role in suppressing epithelial-mesenchymal transition (EMT) [11]. EMT is a process that epithelial cells somehow obtain the properties of mesenchymal cells with the loss of tight cell-cell junction, regulation of cytoskeleton remodeling and increased capability of cell mobility to overcome the restraint of basement-membrane [12]. EMT confers cancerous cells a perfect occasion for metastasizing to distant site with the result of accelerating disease progression. As an assistant of EMT, cytoskeleton rearrangement owns its role in facilitating cell mobility and invasion. And members of Rho GTPase family including RhoA, Rac1, Cdc42 and so on are widely involved in dynamics of actin assembly and disassembly. Among of these members, RhoA has been lucubrated with its downstream target, Rho-associated coiled-coil kinase 1 (ROCK1) and researches have elucidated RhoA/ROCK1 pathway are related to EMT process and tumor metastasis [13, 14]. Similarly, cyclooxygenase 2 (COX2) as an inflammation-related factor and enzyme to catalyze arachidonic acid partly into prostaglandin E_2_ (PGE_2_), exhibits its potential role in disseminating breast cancer cells to distant organs [15]. Meanwhile, PGE_2_ could cause activation of β-catenin, an classic EMT marker of mesenchymal cells, for promoting colon cancer metastasis and COX2/PGE_2_ pathway has also been involved in ovarian cancer angiogenesis [16, 17]. What is more, once binding to a proper ligand, AHR would initiate the transcription of the most well-known targeted downstream genes which include the cytochrome P450-dependent monooxygenases family, such as CYP1A1. Occasionally, I3C has been reported to inhibit AHR binding to the COX2 promoter [4].

Thus, our study aims to explore if DIM could modulate AHR to reverse EMT of ESCC through RhoA/ROCK1 pathway or COX2/PGE_2_ pathway and if these two classic pathways have some interactions synergistically to suppress EMT process and metastasis.

## Methods

### Cell culture and antibodies

We obtained the human ESCC cell lines TE1 and KYSE150 from the Type Culture Collection of the Chinese Academy of Sciences (Shanghai, China). Cells were cultured in RPMI 1640 medium with 10% Fetal Bovine Serum (FBS, Cellmax, China) at an atmosphere of 5% CO_2_ in a humid condition. All antibodies used in Western blot or IHC were shown in Additional file 1: Table S1.

### Immunohistochemistry (IHC) staining

Fifty samples of ESCC patients who had experienced esophagectomy at The First Hospital of China Medical University from 2011 to 2013 were collected for IHC staining. All patients were diagnosed ESCC postoperative pathologically by pathologists and all informed consent letters were signed clearly. Follow-up was conducted by calculating the overall survival (OS) time from date of surgery to date of death or endpoint. Fifty ESCC and paired normal esophagus tissue slides were stained with AHR antibody. Meanwhile, only ESCC slides were treated with RhoA and ROCK1 antibodies. We used H-score for evaluating gene expression levels by combining staining intensity and staining ratio. Staining scores were evaluated as follows: 0 (no staining), 1 (weak), 2 (moderate), 3 (strong). Staining ratio ranged from 0 to 100. H-score (0–300) was calculated through multiplying staining intensity by staining ratio. For survival analysis, cutoff was set by the median of relative protein H-score.

### Bioinformatic analysis

GEPIA (http://gepia.cancer-pku.cn) was conducted for analyzing gene expression levels and correlations between gene A with B of esophageal carcinoma (ESCA) [18]. We downloaded four ESCC databases GSE23400 (53 pairs), GSE38129 (30 pairs), GSE20347 (17 pairs) and GSE29001 (21 ESCC and 24 normal tissues) from GEO Datasets (https://www.ncbi.nlm.nih.gov/geo/). Statistic analysis was conducted with R software 3.2.2. UALCAN (http://ualcan.path.uab.edu) was used for further analyzing the relationships between AHR and clinical pathological parameters [19].

### Wound healing assay

TE1 and KYSE150 cells were treated with various concentrations of DIM or Celecoxib or PGE_2_ dissolved in serum-free medium. Scratches were made using 10 μl pipette tips. Images were acquired by inverted microscope (Leica, Germany) at 0, 24 and 48 h, respectively. We used Image J software (USA) for calculating the relative percentage of wound healing areas.

### Transwell assay

Cells were seeded onto the upper chambers of the Transwell Chamber (8 μm pore size, Costar, USA) with 100 μl serum-free medium containing different concentrations of DIM or Celecoxib or PGE_2_. The lower chambers were filled with 10% FBS medium. After incubation for 24 or 48 h, the upper chambers were removed and fixed with 4% paraformaldehyde (Solarbio, China) for 30 min and then stained with 0.1% crystal violet (Beyotime, China). Images were obtained with the Leica inverted microscope.

### Western blot

TE1 and KYSE150 cell lines were treated with DIM (MCE, USA) at concentrations of 0, 20, 40, 60 μM for 48 h or Celecoxib (MCE, USA) at 60 μM or PGE2 (MCE, USA) at 10 μM or Fasudil (MCE, USA) at 50 μM and 100 μM for 24 h. Cells were harvested with RIPA (Beyotime, China) lysis buffer and then underwent electrophoresis and were transferred onto PVDF membrane (Millipore, USA). Membranes were blocked with QuickBlock™ Blocking Buffer for WB (Beyotime, China) and then incubated with primary antibodies overnight at 4 °C. Secondary antibodies were used and ECL was utilized for visualization.

### Cell morphology

ESCC cells were treated with DIM at 0, 20, 40 and 60 μM concentrations for 48 h. Images of cell morphology changes were captured by invented microscope (Leica, Germany).

### Immunofluorescence of F-actin

ESCC cells were treated with DIM for 48 h or transfection in 12-well plates and after that, cells were washed with PBS and fixed with 4% paraformaldehyde for 30 min. We used 0.5% Triton-X100 to permeate for 15 min and Alexa Fluor 488 Phalloidin (CST, USA) for F-actin staining at a ratio of 1:20 for 30 min. DAPI (Solarbio, China) at the final concentration of 0.5 μg/ml was used for nuclei staining for 15 min. After complete wash, fluorescent images were captured by Live Cell Imaging System (BioTek, USA). Image J was used for fluorescent quantitative analysis.

### Quantitative PCR (qPCR)

Total RNA was extracted from treated cells using the miRNeasy Mini Kit (QIAGEN, Germany) and quantified with spectrophotometer (BioTek, USA). cDNA was synthesized with GoScript Reverse Transcription Kit (Promega, USA). qPCR was performed with GoTaq qPCR System Kit (Promega, USA) using the 7900HT qPCR System (Applied Biosystems, USA). The primer sequences were listed in Additional file 1: Table S2.

### Elisa

To detect relative expression levels of PGE_2_ after treatment of various concentrations of DIM dissolved in serum-free medium incubating for 48 h, ELISA was performed by collecting supernates respectively. The PGE_2_ released into the medium was measured using the PGE_2_ ELISA Kit (R&D System, USA) and all procedures were in agreement with corresponding protocols.

### Cell transfection

ESCC cells were transfected with lentivirus (Genechem, China) or siRNAs (OriGene, USA). For AHR transfection, shRNA 1 sequence was used as GCATAGAGACCGACTTAAT and shRNA 2 as AACAAGATGAGTCTATTTA. For ROCK1, siRNA 1 sequences were 5′-CGGUUAGAACAAGAGGUAAAUGAAC-3′ and siRNA 2 were 5′-GGAAAUAUCAAACGAUAUGGCUGGA-3′. For PTGS2 (COX2), siRNA 1 squences were 5’GGCUAAUACUGAUAGGAGAGACUAT-3′ and siRNA 2 were 5′-GCAGCUUCCUGAUUCAAAUGAGATT-3′. Meanwhile, overexpression of AHR (OE-AHR) with lentivirus (Genechem, China) in TE1 and KYSE150 cell lines was also conducted with sequence as GAGGATCCCCGGGTACCGGTCGCCACCATGAACAGCAGCAGCGCCAACATC.

### Animal study

The animal study was approved by the Animal Center of China Medical University (No.2018146). Four to six weeks old BALB/C nude male mice were purchased from Beijing Vital River Laboratory (Beijing, China) and raised in condition of SPF level. Sixteen nude mice were inoculated subcutaneously with approximately 2 × 10^6^ TE1 cells and randomly separated into two groups (Control and DIM groups) after 1 week. Control group was fed with PBS while DIM group was treated with DIM (10 mg/kg/day). After 4 weeks of gavage, mice were sacrificed and xenograft tumors were removed to prepare for IHC staining. Image J was used for quantitive analysis.

### Co-immunoprecipitation (co-IP) assay

Proteins were harvested after certain treatment and divided into two parts, one for co-IP and the other for Input assay. Approximate 200 μl protein lysates were incubated with 40 μl Protein A + G Agarose (Beyotime, China) and proper volume of antibodies (Additional file 1: Table S1) as well as Rabbit IgG antibody (Beyotime, China) at 4 °C overnight with gentle shaking. Then, samples were centrifuged at 6000 rpm at 4 °C for 3 min and washed with 1 ml pre-cooling PBS for at least five times repeatedly. About 10 μl 5 × SDS was added into each tube after resuspended with 40 μl PBS and samples were heated to 95 °C for 10 min. Finally, samples were loaded on 10% SDS-PAGE for WB analysis.

### Statistical analysis

All experiments were repeated at least three times and data were shown as mean ± standard deviation (SD). SPSS 20 and GraphPad Prism 8 were used for analyzing data and creating statistical graphics. For comparing statistical significance of two groups, paired Student’s t-test was used and Spearman correlation analysis was used for comparing relationships between genes of ESCC. For comparisons of more than three groups, one-way ANOVA was used and LSD method was used for multiple comparisons. Mann-Whitney test was used for analyzing the correlations between H-scores and clinical pathological parameters. Kaplan-Meier method and Cox regression analysis were performed for ESCC prognostic analysis. A *P*-value less than 0.05 was considered statistical significant.

## Results

### AHR is highly expressed in ESCC and correlates with poor clinical pathological parameters

Results of IHC H-scores showed that AHR was highly expressed in ESCC compared with normal tissues (Additional file 2: Fig.S1A and 1B) and significantly correlated with TNM stage and lymph nodes metastasis (Additional file 2: Fig.S1C and 1D), while it had no significant associations with patients’ age, smoking habit, tumor differentiation and T stage (data not shown). AHR expression levels were in line with the degree of lymph node metastasis (Additional file 2: Fig.S1E). GEPIA database indicated that AHR expression levels were actually elevated in esophageal carcinoma (ESCA) compared with normal tissues (Additional file 2: Fig.S1F). We employed 4 ESCC databases downloaded from GEO Datasets and results showed that AHR were upregulated in all these 4 databases (Additional file 2: Fig.S1G). UALCAN database showed AHR expression levels significantly correlated with tumor grade, lymph node metastasis and clinical stages (Additional file 2: Fig.S1H).

### DIM inhibits migration and invasion by reversing EMT of ESCC

**Fig. 1:**
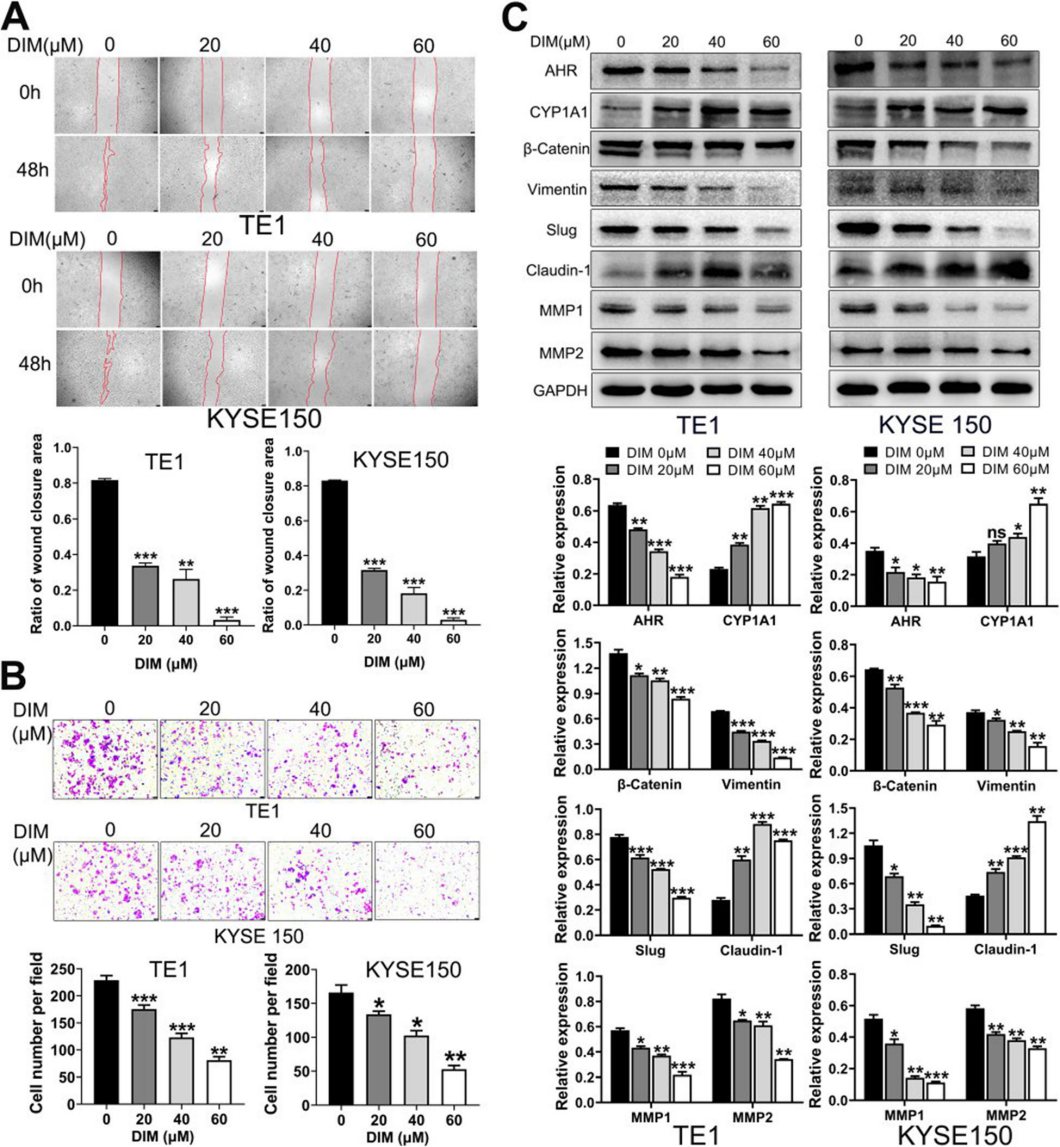
DIM can inhibit ESCC migration and invasion by reversal of EMT through modulation of AHR. **a**. Wound healing assay showed decreased ability of migration of both TE1 and KYSE150 cells. **b**. Transwell assay indicated DIM could inhibit invasion in a dose-dependent manner. **c**. Western blot implied that DIM could modulate AHR to reverse EMT process with downregulation of mesenchymal cell marker β-Catenin, Vimentin and Slug as well as MMPs and upregulation of epithelial cell marker Claudin-1. * *P* < 0.05, ** *P* < 0.01, *** *P* < 0.001, ns, no significance

We first examined the expression levels of AHR in ESCC cell lines. Results showed that AHR was at moderate or high expression level in TE1 or KYSE150 cell line (Additional file 2: Fig.S2). Since AHR was overexpressed in ESCC, we treated cells with AHR modulator DIM to further elucidate the effect of AHR on ESCC metastasis. As shown in Fig. 1a and b, TE1 and KYSE150 were treated with various concentrations of DIM and wound healing assay and Transwell assay indicated the capabilities of cell migration and invasion were weakened in a dose-dependent manner. WB results demonstrated that DIM could modulate AHR via shifting AHR into the nuclei to participate transcription activity with the symbol of increasing levels of CYP1A1 production along with the elevated concentrations of DIM. Meanwhile, DIM could reverse the EMT process of ESCC with downregulated expression levels of mesenchymal cell marker β-Catenin, Vimentin and Slug, and in contrast, upregulated that of epithelial cell marker Claudin-1. What is more, DIM could also inhibit expression of MMP1, and MMP2 (Fig. 1c).

### DIM can rearrange ESCC cytoskeleton by disassembling F-actin through RhoA/ROCK1 pathway

**Fig. 2:**
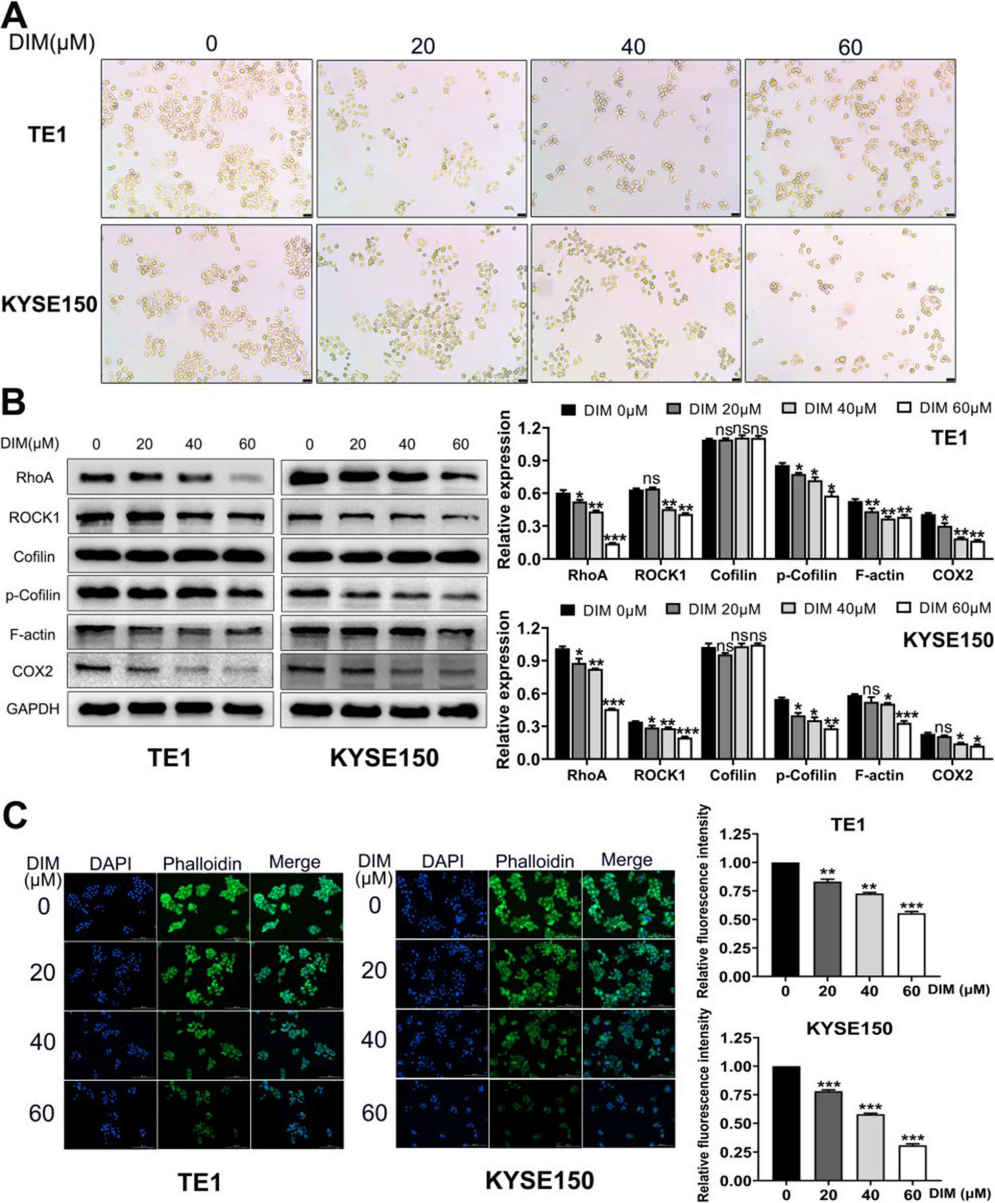
DIM can induce ESCC rearrangement of cytoskeleton through RhoA/ROCK1 pathway. **a**. ESCC cell morphology had changed from irregular long fusiform to almost round or ellipse after DIM treatment. **b**. WB showed DIM could downregulate the cytoskeleton-associated RhoA/ROCK1/Cofilin pathway to decrease expression of F-actin. **c**. Phalloidin staining of F-actin visualized the relative expression levels after various concentrations of DIM treatment. * *P* < 0.05, ** *P* < 0.01, *** *P* < 0.001, ns, no significance

After DIM treatment, we found that ESCC cell morphology changed from irregular long fusiform to almost round or ellipse which was opposite to EMT process (Fig. 2a). And RhoA/ROCK1/F-actin pathway was reported to correlate with maintaining dynamic balance of actin assembly and disassembly. Therefore, We performed WB to detect pathway-associated proteins change. Results were as follows: RhoA and ROCK1 expression levels were decreased in a dose-dependent manner and so did the expression of p-cofilin and F-actin. Meanwhile, since I3C could inhibit AHR binding to COX2 promoter, we also detected COX2 levels and result showed that DIM could also decrease COX2 expression (Fig. 2b). F-actin was visualized with phalloidin staining and we found F-actin was indeed gradually decreased with elevated concentrations of DIM (Fig. 2c).

### RhoA and ROCK1 are overexpressed in ESCC and positively correlate with lymph node metastasis, stage and AHR expression

**Fig. 3:**
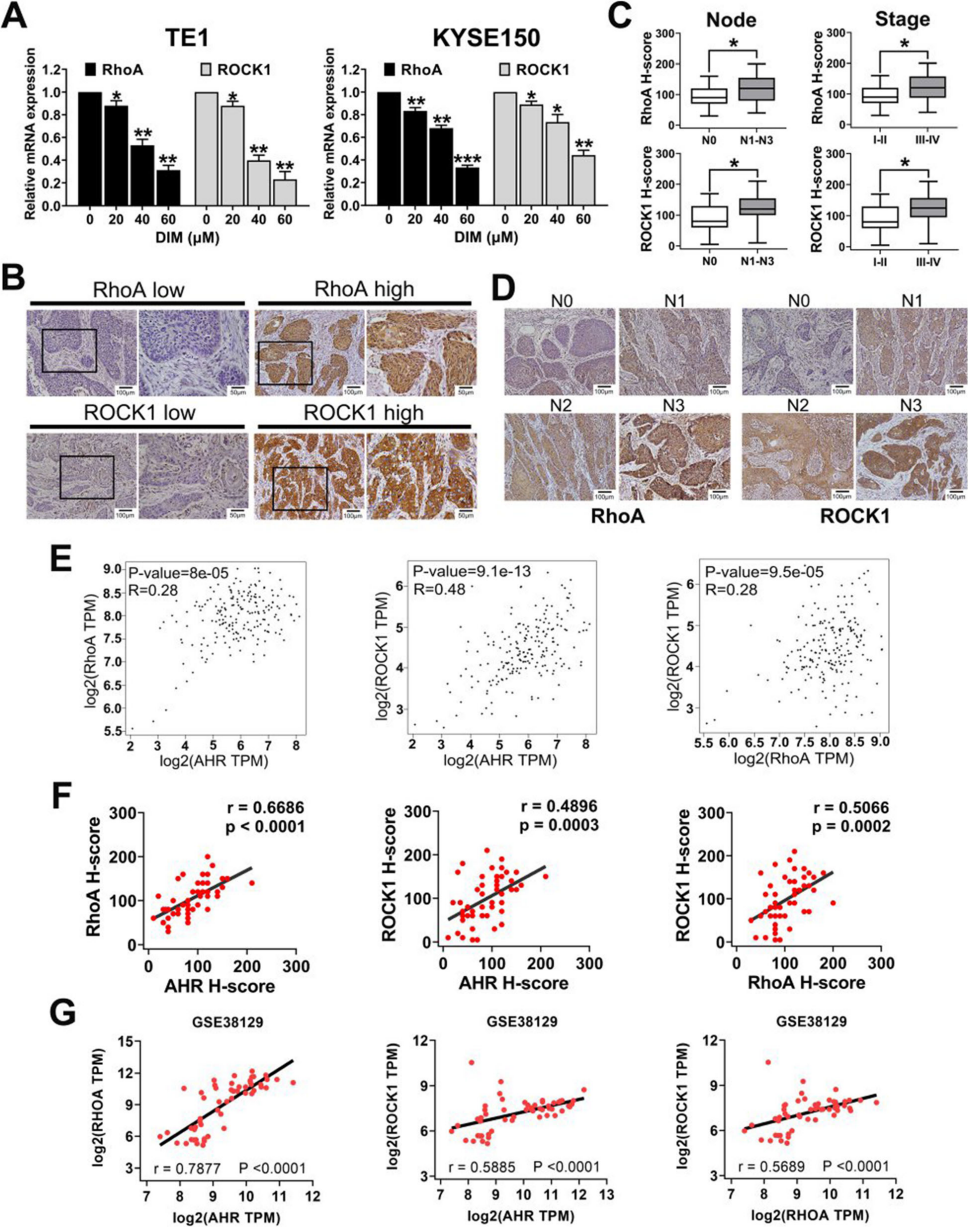
RhoA and ROCK1 in ESCC correlated with poor clinical outcomes and AHR expression levels. **a**. DIM could inhibit transcription of RhoA and ROCK1 in a dose-dependent manner. **b**. Representative IHC images of RhoA and ROCK1 expression levels in ESCC. **c**. RhoA and ROCK1 expression levels significantly correlated with lymph node metastasis and clinical stage. **d**. IHC staining-intensities positively correlated with levels of N stage. **e**. GEPIA database showed significant correlations among AHR, RhoA and ROCK1 in ESCA. **f**. Correlation analysis with IHC staining exhibited the same significant results as GEPIA. **g**. GSE38129 database verified that positive correlation among AHR, RhoA and ROCK1 expression levels in ESCC. * *P* < 0.05, ** *P* < 0.01, *** *P* < 0.001, ns, no significance

Since DIM could rearrange ESCC cytoskeleton through RhoA/ROCK1 pathway, we first examined the relative mRNA expression levels of RhoA and ROCK1 by qPCR. Results indicated that DIM could also suppress the transcription levels of RhoA and ROCK1 (Fig. 3a). Next, we wondered whether these two genes were related with poor clinical outcome of ESCC. Therefore, we used IHC to detect expressions of RhoA and ROCK1 in ESCC samples which were consistent with those of AHR staining. After data reduction and analysis, results showed the low and high expression of RhoA and ROCK1 in Fig. 3b, and both RhoA and ROCK1 levels were significantly associated with lymph node metastasis and clinical stages (Fig. 3c). Along with the N stage exacerbation, RhoA and ROCK1 staining intensities were enhanced (Fig. 3d). With that, we utilized the GEPIA database to explore the correlations between AHR and RhoA or ROCK1 in ESCA and we found AHR gene was positively correlated with RhoA and ROCK1 gene (Fig. 3e). Through analysis of these three genes expression levels of the same ESCC slide, the results were same as GEPIA analysis (Fig. 3f). Finally, we performed the aforesaid analysis of the four GEO databases. However, only GSE38129 database completely exhibited the same results (Fig. 3g) and the other three databases showed no significant correlations except for part of the analysis (Additional file 2: Fig. S3).

### Synergic overexpression of AHR, RhoA and ROCK1 predicts poor prognosis and blockade of RhoA/ROCK1 pathway reverses EMT

**Fig. 4:**
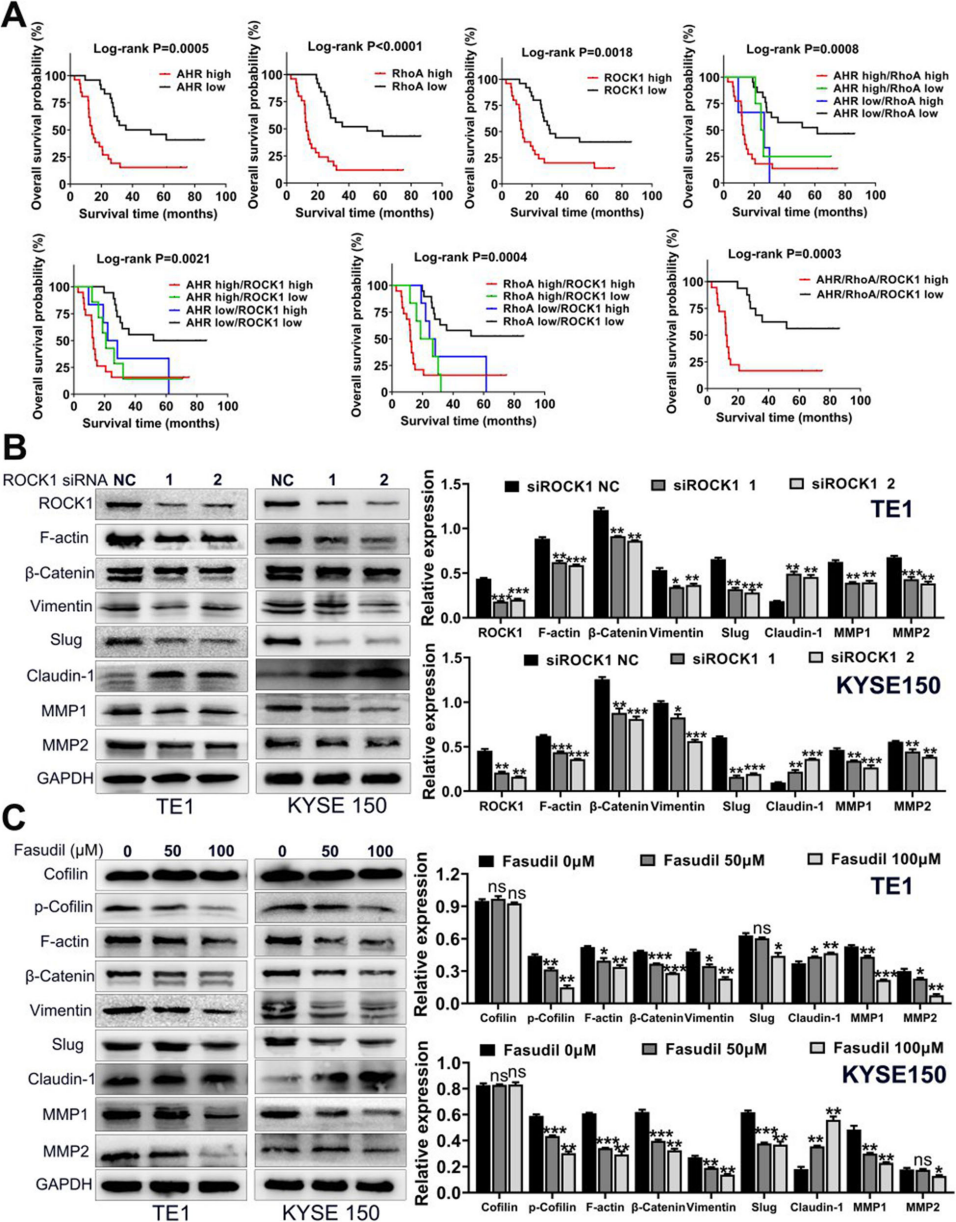
Co-overexpression of AHR, RhoA and ROCK1 predicted poor prognosis and targeting RhoA/ROCK1 pathway can reverse EMT. **a**. Survival analysis of single AHR, RhoA or ROCK1 expression levels or combined. Co-overexpression of above three proteins predicted poor prognosis of ESCC. **b**. Knockdown of ROCK1 with specific siRNAs could reverse EMT process as well as downregulate expression of MMPs. **c**. Blockade of RhoA/ROCK1 pathway with inhibitor Fasudil could actually reverse EMT of ESCC. * *P* < 0.05, ** *P* < 0.01, *** *P* < 0.001, ns, no significance

Since AHR, RhoA and ROCK1 were all associated with lymph node metastasis and clinical stage, and they correlated significantly with each other, we wondered if they had any relationships with overall survival time. Thus, we performed the survival analysis of AHR, RhoA and ROCK1 independently or in combination. Results showed individual overexpression of AHR, RhoA or ROCK1 predicted poor prognosis. What was more, when two of the three genes or all three genes were co-overexpressed, the overall survival probability was much lower compared with low expression group (Fig. 4a). Meanwhile, we conducted multivariate Cox regression analysis of prognostic factors regarding AHR, RhoA and ROCK1 expression levels. Results in Table 1 demonstrated that all these three proteins were significant risk factors for ESCC prognosis [AHR HR: 1.010(1.001–1.020), *P* = 0.038; RhoA HR: 1.012(1.003–1.020), *P* = 0.010; ROCK1 HR: 1.009(1.001–1.018), *P* = 0.031]. It signified the conjecture that AHR, RhoA and ROCK1 were all associated with EMT process in order to facilitate progression of ESCC. Hence, we next examined whether ROCK1 correlated with EMT through ROCK1 specific small interfering RNA (siRNA). As shown in Fig. 4b, after knockdown of ROCK1, F-actin and mesenchymal cell markers as well as MMP1 and MMP2 expression levels were decreased while epithelial cell marker Claudin-1 was upregulated. Phalloidin staining of F-actin treated with ROCK1 siRNAs was in line with WB (Additional file 2: Fig. S4). Meanwhile, we used the RhoA/ROCK1 pathway inhibitor Fasudil to further verify the effect of this pathway on EMT. Data indicated that through inhibiting the pathway, Fasudil could also reverse EMT process (Fig. 4c). In this way, blockade of RhoA/ROCK1 pathway could inhibit ESCC progression.

**Table 1:** Cox regression for prognostic analysis with IHC results of ESCC

Variables	β	SE	Wald	df	*P* value	HR	95%CI
AHR	0.010	0.005	4.286	1	0.038	1.010	1.001–1.020
RhoA	0.011	0.004	6.611	1	0.010	1.012	1.003–1.020
ROCK1	0.009	0.004	4.644	1	0.031	1.009	1.001–1.018

*β* regression coefficient; *SE* standard error; *Wald* wald chi-square; *df* degree of freedom; *HR* hazard ratio; *CI* confidence interval

### COX2/PGE_2_ pathway correlates with ESCC migration and invasion

**Fig. 5:**
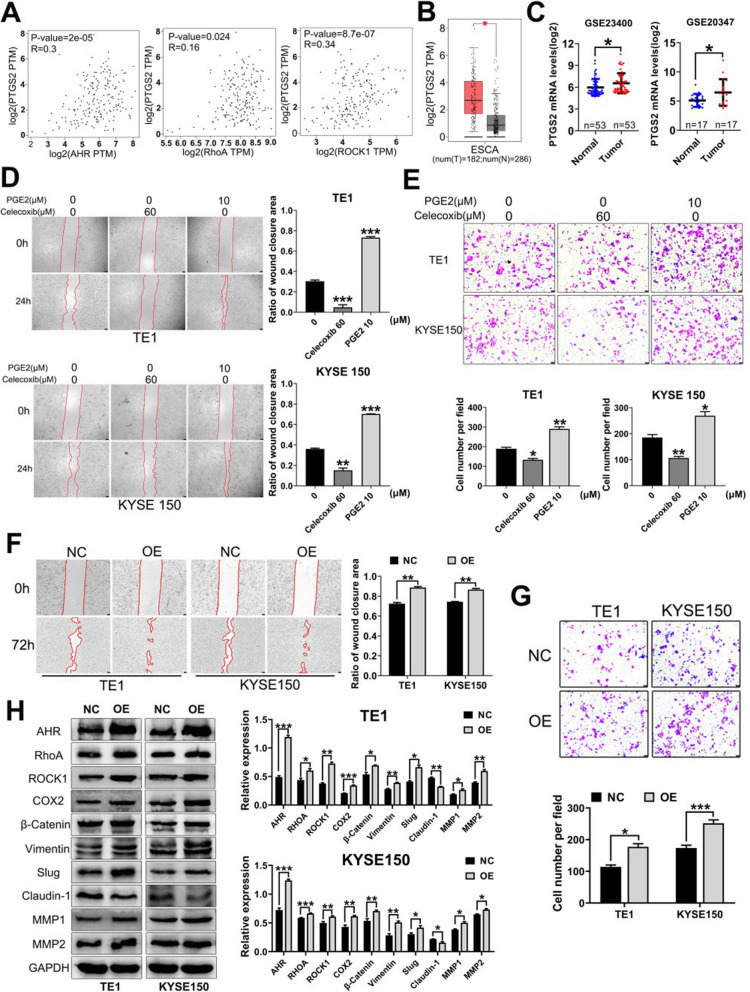
Targeting COX2/PGE_2_ pathway affects ESCC migration and invasion and overexpression of AHR promotes EMT process. **a**. GEPIA database showed positive correlations between PTGS2 (COX2) and AHR or RhoA or ROCK1. **b**. GEPIA database showed PTGS2 was overexpressed in ESCA. **c**. GSE23400 and GSE20347 verified PTGS2 expression levels in ESCC were elevated. **d** and **e**. Wound healing assay exhibited after COX2 selective inhibitor Celecoxib treatment, cell abilities of migration and invasion were inhibited while after PGE_2_ treatment, the abilities were strengthened. **f** and **g**. Overexpression of AHR could strengthen ESCC migration and invasion. H. Overexpression of AHR could promote EMT process. * *P* < 0.05, ** *P* < 0.01, *** *P* < 0.001, ns, no significance

As mentioned above, DIM could downregulate COX2 expression. Then we tried to verify whether COX2/PGE_2_ pathway was involved in EMT process of ESCC. From GEPIA database, we noticed PTGS2 (gene name of COX2) expression levels were significantly positively related with AHR, RhoA and ROCK1 in ESCA (Fig. 5a). Moreover, COX2 was also overexpressed in ESCA (Fig. 5b) and through analysis of GEO databases, GSE23400 and GSE20347 databases indicated overexpression of COX2 (Fig. 5c) while the other two showed no significance (Additional file 2: Figure S5). Since evidence showed COX2 was aberrantly expressed in ESCC, we aimed to test the function of COX2/PGE_2_ pathway regarding EMT with the use of COX2 selective inhibitor Celecoxib and catalysate PGE_2_. Wound healing assay and Transwell assay exhibited that Celecoxib could suppress TE1 and KYSE150 cells migration and invasion while after PGE_2_ treatment, ESCC migratory and invasive abilities were strengthened (Fig. 5d and e).

### Overexpression of AHR promotes EMT process with increased capacity of migration and invasion

Since we aimed to explore the underlying mechanism of reversing EMT process through modulation of AHR, we next constructed stable transfected cell lines of AHR overexpression (OE-AHR) to verify the proper phenotype change and pathway. As shown in Fig. 5f and g, overexpression of AHR promoted ESCC migration and invasion. WB results indicated that after overexpression of AHR, RhoA/ROCK1 and COX2 expression levels were also elevated. Meanwhile, EMT process was actually promoted with upregulated expression of mesenchymal cell markers and downregulated that of epithelial cell marker Claudin-1 (Fig. 5h).

### DIM targets COX2/PGE_2_ pathway to reverse EMT

**Fig. 6:**
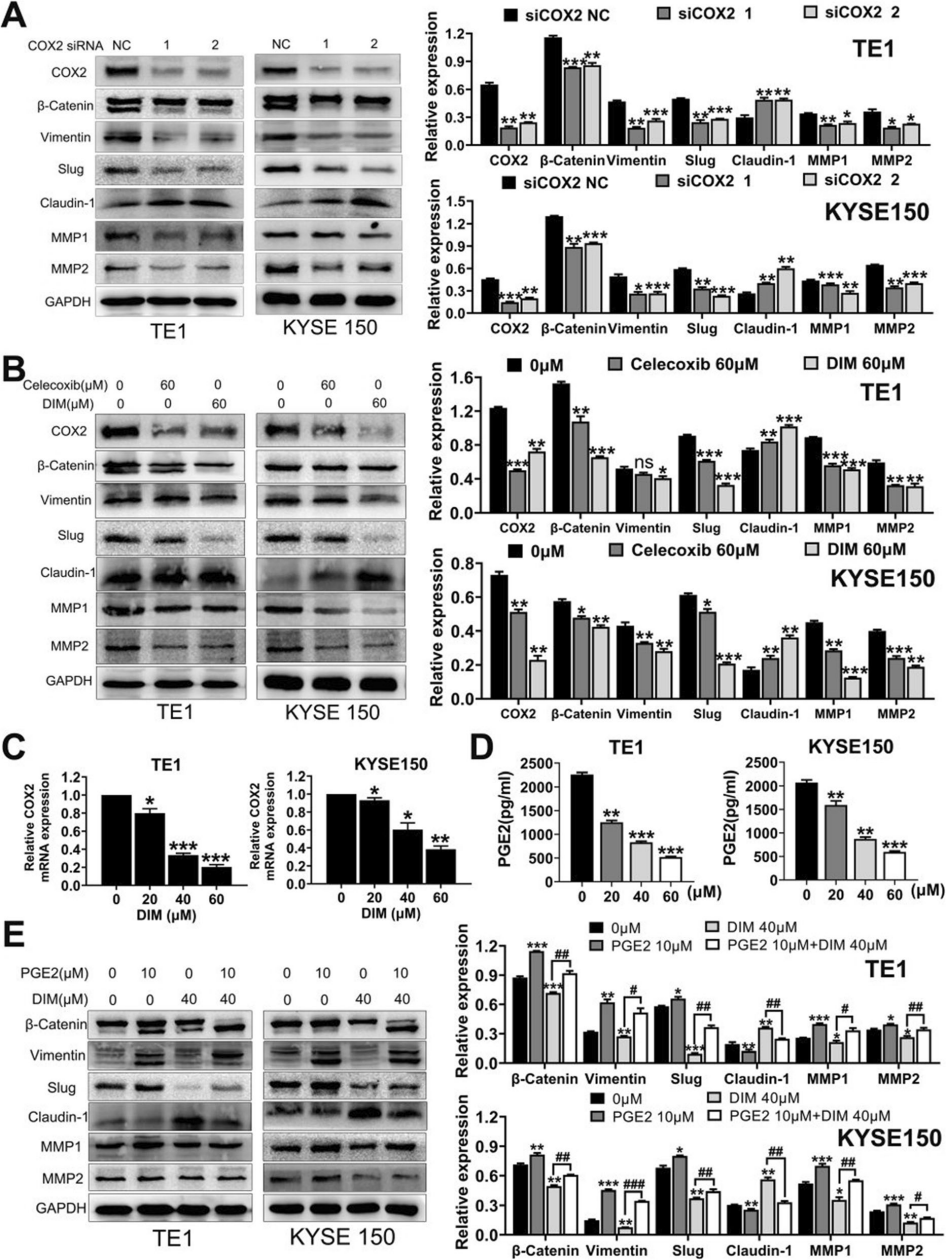
Targeting COX2/PGE_2_ pathway affects EMT process of ESCC. **a**. Knockdown of COX2 with specific siRNAs could reverse reverse EMT process with downregualtion of β-Catenin, Vimentin and Slug as well as MMPs and upregulation of Claudin-1. **b**. COX2 selective inhibitor Celecoxib synergically with DIM inhibited EMT. **c**. DIM inhibited transcription of COX2 measured by qPCR. **d**. DIM inhibited production of PGE_2_ in a dose-dependent manner measured by ELISA assay. **e**. WB results showed that DIM could partly reverse the EMT process which could be enhanced by PGE2 treatment. * *P* < 0.05, ** *P* < 0.01, *** *P* < 0.001, ns, no significance

Since COX2/PGE_2_ pathway was involved in tumor metastasis, we utilized COX2 specific siRNAs and celecoxib as well as PGE_2_ to further explore its relationship with EMT process. As shown in Fig. 6a, after treated with COX2 siRNAs, TE1 and KYSE150 cells exhibited downregulated expression levels of β-Catenin, Vimentin, Slug, MMP1 and MMP2, and upregulated Claudin-1 expression. The results of celecoxib treatment were similar to that of COX2 siRNAs treatment (Fig. 6b). To verify WB alterations of COX2 expression after DIM treatment, we next examined the COX2 mRNA levels changes by qPCR. As expected, DIM could inhibit COX2 relative mRNA expression levels in a dose-dependent manner (Fig. 6c). As a matter of course, we then used ELISA assay to detect the levels of PGE_2_ and results were consistent with the COX2 expression levels after DIM incubation (Fig. 6d). Thus, we directly added PGE_2_ in medium to examine relative proteins alterations. WB results indicated PGE_2_ could exacerbate EMT process while DIM could actually reverse EMT in part (Fig. 6e). Through targeting COX2/PGE_2_ pathway, DIM could reverse EMT of ESCC.

### DIM modulates AHR to reverse EMT through repressing RhoA/ROCK1-mediated COX2/PGE_2_ pathway

**Fig. 7:**
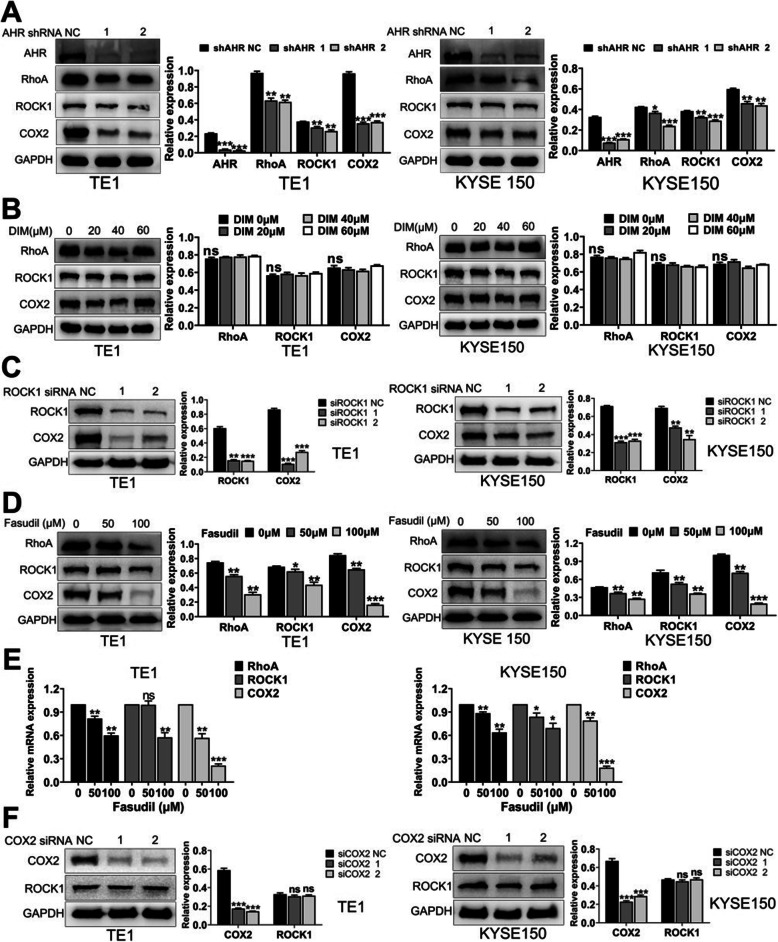
COX2/PGE_2_ pathway can be mediated by RhoA/ROCK1 pathway. **a**. Knockdown of AHR inhibited expression levels of RhoA, ROCK1 and COX2. **b**. After knockdown of AHR, DIM exerted no obvious effect on RhoA/ROCK1 and COX2 expression. **c**. Knockdown of ROCK1 inhibited expression of COX2. **d**. Inhibition of RhoA/ROCK1 pathway with Fasudil could suppress COX2 expression. **e**. Results by qPCR indicated Fasudil could inhibit transcription of RhoA/ROCK1 and COX2 expression in a dose-dependent manner. **f**. Knockdown of COX2 had no effects on ROCK1 expression levels. * *P* < 0.05, ** *P* < 0.01, *** *P* < 0.001, ns, no significance

After elucidating the fact that RhoA/ROCK1 and COX2/PGE_2_ pathway were involved in EMT process of ESCC, we wondered if these two pathways had some interactions in regulating cytoskeleton and EMT process, and whether they were related with AHR. Therefore, we established AHR knockdown stable transfection cell lines with lentivirus to examine related proteins alterations. WB results demonstrated that after knockdown of AHR, all RhoA, ROCK1 and COX2 expression levels were synergically decreased (Fig. 7a). Similarly, we treated sh-AHR cells with same concentrations of DIM and no clear alterations of RhoA, ROCK1 and COX2 were shown, Which represented that AHR was the upstream gene of RhoA/ROCK1 and COX2/PGE_2_ pathway (Fig. 7b). Next, we used ROCK1 siRNAs and RhoA/ROCK1 pathway inhibitor Fasudil to treat ESCC cells and results exhibited after knockdown of ROCK1 or inhibition of the pathway, COX2 protein expression levels were decreased in the same way (Fig. 7c and d). Meanwhile, qPCR showed that Fasudil could actually downregulate RhoA, ROCK1 and COX2 relative mRNA levels (Fig. 7e). Finally, after knockdown of COX2 with siRNAs, there were no clear alterations of ROCK1 protein expression levels, which represented COX2 was the downstream gene of ROCK1 (Fig. 7f). To summarize, DIM modulated AHR to reverse EMT through repressing RhoA/ROCK1-mediated COX2/PGE_2_ pathway.

### In vivo experiment verifies DIM can reverse EMT process of ESCC

**Fig. 8:**
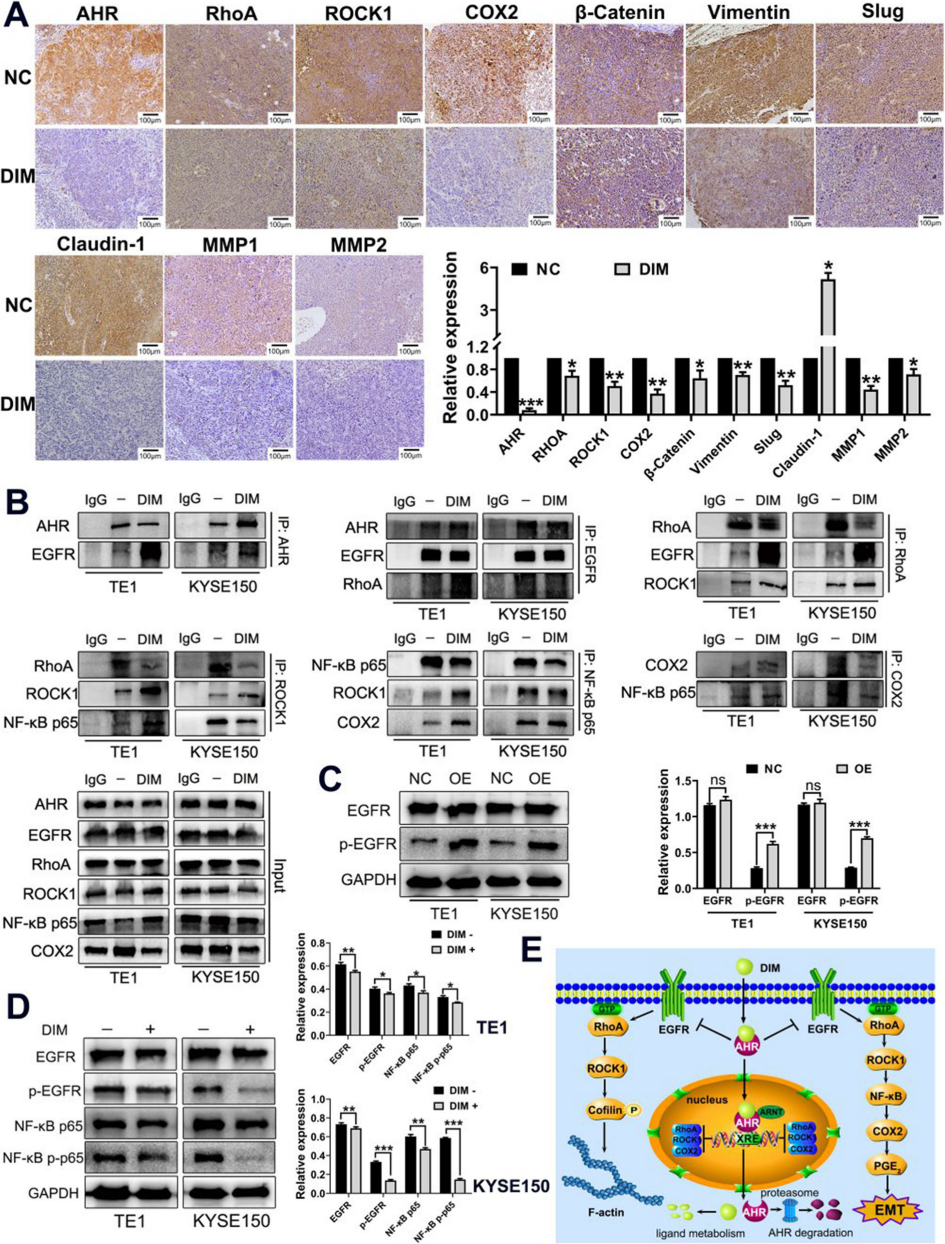
In vivo assay and co-IP assay verifies that DIM can modulate AHR to reverse EMT through repressing RhoA/ROCK1-mediated COX2/PGE_2_ pathway. **a**. IHC results of xenografts staining showed downexpression of AHR, RhoA, ROCK1, COX2 and mesenchymal cell markers as well as upregulation of epithelial cell marker Claudin-1. **b**. Co-IP assay indicated that proteins had direct interactions between each other. DIM+: 40 μM. **c**. Overexpression of AHR resulted in elevated levels of p-EGFR. **d**. DIM (40 μM) could inhibit expressions of EGFR and p-EGFR as well as NF-κB p65 and p-p65. **e**. Schematic representation of molecular mechanism of DIM in reversal of EMT process of ESCC

We performed in vivo experiment on BALB/C nude mice treated with TE1 cells injection subcutaneously and DIM gavage to verify the in vitro results. As expected, IHC staining results showed downregulated expression levels of AHR, RhoA, ROCK1, COX2 and mesenchymal cell markers as well as MMP1 and MMP2. Epithelial cell marker Claudin-1 was upregulated (Fig. 8a). All in vivo results were in line with in vitro ones.

### Modulation of AHR by DIM leads to weakened protein-protein interaction with EGFR to suppress RhoA/ROCK1-mediated COX2/PGE2 pathway connected by NF-κB

Even though we had certified the proper underlying mechanism, we wondered whether these proteins had any interactions between each other. However, we had not obtained the direct interaction except for that between RhoA and ROCK1 (data not shown). Therefore, we proposed that AHR might have interaction with EGFR so as to regulate downstream pathways after carefully reviewing documents. As shown in Fig. 8b, we found the direct interaction between AHR and EGFR with co-IP assay. What was more, EGFR and NF-κB had been reported to have associations with RhoA/ROCK1 and COX2/PGE_2_ pathway respectively. NF-κB had also been detected to exhibit direct interactions with ROCK1 and COX2. To further determine the effective interaction between AHR and EGFR, we utilized AHR overexpression cell lines for detection level changes of EGFR and p-EGFR. Results verified that p-EGFR expression levels were elevated while expressions of total EGFR remained unchanged (Fig. 8c). Similarly, DIM could inhibit expression levels of EGFR and p-EGFR as well as NF-κB p65 and p-p65 (Fig. 8d). To sum up, DIM could modulate AHR to transfer into the nuclei for participation in transcription activity and to weaken interaction with EGFR leading to inhibition of RhoA/ROCK1-mediated COX2/PGE_2_ pathway connected by NF-κB to finally reverse EMT process of ESCC (Fig. 8e).

## Discussion

Esophageal squamous cell carcinoma is one of the most aggressive tumors with rapid tumor progression and lower five-year survival rate. There are still no effective clinical drugs that can block the metastatic process, especially of ESCC patients at advanced stage [20, 21]. For metastasis, a novel process is described as follows: cancer cells obtain the capability of disseminating from primary tumor site to proper distant organs with strengthened migratory and invasive abilities. Epithelial-mesenchymal transition is a complicated program that it confers epithelial cells successful transformation into mesenchymal cells with morphological change which leads to attenuated cell-cell junction, dissolved cell-matrix adhesion, rearranged cytoskeleton and enhanced invasive growth [12]. EMT program is the vital initiation of tumor metastasis and targeting EMT as one of the most effective solutions to block ESCC progression is deserved explorations. Aryl hydrocarbon receptor is a ligand-dependent transcription factor which has been reported to have connections with tumor metastasis of thyroid carcinoma, neuroblastoma and inflammatory breast cancer [22–24]. And DIM as a selective modulator of AHR, has been demonstrated it has inhibitory effects on tumor proliferation, migration and invasion [25, 26]. Meanwhile, our previous study have demonstrated that AHR overexpression contributed to worse functional phenotypes and DIM treatment could inhibit ESCC cell growth, induce G1 phase arrest and promote cell apoptosis of TE1 cells [10]. Therefore, we want to explore whether DIM can reverse EMT process of ESCC through modulation of AHR. As expected, DIM actually can downregulate expressions of mesenchymal cell markers including β-Catenin, Vimentin and Slug and epithelial cell marker Claudin-1 has been upregulated. Meanwhile, DIM also exerts its prohibitive effects on cell migration and invasion.

For EMT process, we have identified rearrangement of cancer cytoskeleton emphasizes its role in EMT and from experimental observation, cellular morphology of ESCC has changed from irregular long fusiform to almost round or ellipse type which implies the disruption of dynamic balance of local assembly and disassembly of cell actin filaments. Cytoskeleton regulation is the prerequisite of cell endocytosis, mobility, migration and invasion [27]. RhoA/ROCK1 pathway has been reported to regulate actin stress fiber formation, cell-cell junction and cell-matrix adhesion by inducing MMPs production or regulating CXCR4/Akt signaling [28, 29]. What is more, overexpression of RhoA or ROCK1 has contributed to malignant phenotype of cancers, such as ESCC [30]. DLC1 SAM domain-binding peptides have been reported to inhibit breast cancer growth and migration through inactivation of RhoA [31] and ROCK1 can promote migration and invasion of non small cell lung cancer by activating PTEN/PI3K/FAK pathway [32]. Our results verify that DIM can reduce F-actin assembly through inhibition of RhoA/ROCK1 pathway with decreased transcription activities and kinase activities. Evidences show that AHR can bind to the XREs in the RhoA promoter region at the position of − 455 to − 431 bp with AHR agonist 3-MC (3-methylcholanthrene) [33] and EGFR has been reported to promote human trophoblast cell migration through activation of RhoA and RhoC [34]. Moreover, EGFR has been reported to interact with AHR and GPER (G protein estrogen receptor) to stimulate breast cancer progression and EGFR can bypass RhoA to activate YAP signaling to promote hepatocellular carcinoma proliferation [35, 36]. Meanwhile, our co-IP results showed direct protein-protein interactions between AHR and EGFR as well as EGFR and RhoA. Thus, we hypothesize that DIM can modulate AHR, which leads to decreased transcription activity of RhoA/ROCK1 pathway and weakened interaction with EGFR since overexpression of AHR contributes to elevated expression levels of p-EGFR. Certainly, F-actin assembly or disassembly makes sense for cell migration with involvement in formation of two distinguishing functional states that mediate cell protrusive and contractile steps to couple with the extracellular matrix (ECM) for further generation of MMPs [37]. As reported, vitamin C can inhibit triple-negative breast cancer metastasis by suppressive effect on formation of F-actin and lamellipodia through regulating expression of YAP1 and synaptopodin 2 [38]; Migration and invasion enhancer 1 (MIEN1) has recently been indicated to promote cancer progression and metastasis by polymerizing G-actin and stabilizing F-actin filaments [39].

As mentioned above, DIM can block transcriptional activity of AHR binding to the COX2 promoter. COX2/PGE_2_ pathway is associated with tumor EMT and metastasis. Inhibition of COX2/PGE_2_ pathway can effectively suppress tumor growth, EMT and metastasis of non small cell lung cancer or extrahepatic cholangiocarcinoma through PLA2G4A/PGE_2_/STAT3 pathway [40, 41]. Our results show that DIM can inhibit expression of COX2 and PGE_2_ and COX2 selective inhibitor Celecoxib limits the capabilities of ESCC migration and invasion as well as reverses EMT process. On the contrary, directly supplying ESCC with PGE_2_ promotes EMT and metastasis. Since both RhoA/ROCK1 pathway and COX2/PGE_2_ pathway are able to reverse EMT and inhibit metastasis regulated by modulation of AHR by DIM, we wonder whether some interactions exist. As reported, salidroside can regulate Rho/ROCK1/NF-κB pathway to ameliorate arthritis-induced brain cognition deficits and microRNA-145 can inhibit proliferation and promote apoptosis of hepatocellular carcinoma through downregulation of ROCK1/NF-κB pathway [42, 43]. It is well-known that NF-κB can regulate COX2/PGE2 pathway and is the upstream regulator of the pathway. Thus, we hypothesize that NF-κB is the connection of RhoA/ROCK1 and COX2/PGE2 pathways. Our co-IP assay had demonstrated that NF-κB actually had direct interactions with ROCK1 and COX2. Meanwhile, DIM can also inhibit transcription activity of NF-κB and phosphorylation level. That is to say, DIM exerts its reversal of EMT process mainly through modulation of AHR to inhibit EGFR/RhoA/ROCK1/NF-κB/COX2/PGE_2_ pathway. This is the first time for us to demonstrate that AHR is the upstream gene of above pathways and COX2/PGE_2_ pathway can be mediated by RhoA/ROCK1 pathway, which stands a chance to investigation of ESCC targeted therapy. Therefore, blockade of AHR as the original source of EMT process in ESCC with DIM as the modulator is of significance. Through reversal of EMT process that is currently in the limelight of keeping cancer cells in captivity, we can finally anchored tumors at primary sites to achieve success of prolonged patients’ lifespan.

## Conclusions

In brief, our study indicates that DIM can modulate AHR leading to decreased transcription activities of relative genes and weakened interaction with EGFR to effectively reverse EMT process of ESCC with rearrangement of cytoskeleton and regulation of related EMT markers as well as MMPs through repressing RhoA/ROCK1-mediated COX2/PGE_2_ pathway which is connected by NF-κB. AHR can be explored as a promising target gene and DIM may be applied in clinical treatment of ESCC in the future.

## Supplementary information

**Additional file 1 Table S1.** Information of antibodies used in WB or co-IP **Table S2.** Sequences of primer used in qPCR

**Additional file 2 Supplementary Figure 1**. AHR is overexpressed in ESCC and correlates with poor clinical outcomes. A. IHC results showed represented images of AHR expression levels in ESCC. B. AHR was overexpressed in ESCC compared with paired normal tissues. Expression levels of AHR correlated with ESCC clinical stages (C) and lymph node metastasis (D). E. AHR staining-intensities were positively associated with levels of lymph node metastasis. F. GEPIA database indicated overexpression of AHR in ESCA. G. All four ESCC databases downloaded from GEO Dataset verified AHR was overexpressed in ESCC. H. UALCAN database indicated AHR expression levels were significantly associated with tumor histology, tumor grade, lymph nodal metastasis status and clinical stages. * *P* < 0.05, ** *P* < 0.01, *** *P* < 0.001. **Supplementary Figure 2.** Detection of AHR expression levels in ESCC cell lines by WB. **Supplementary Figure 3.** Correlation analysis of AHR, RhoA and ROCK1 with GEO Datasets. A: In GSE23400 databases, no significant correlations were found among AHR, RhoA and ROCK1 expression levels. B: In GSE20347, only correlation analysis of AHR and ROCK1 was significant. C: In GSE29001, only correlation analysis of RhoA and ROCK1 was significant. **Supplementary Figure 4.** Phalloidin staining of F-actin in TE1 (A) and KYSE150 (B) cell lines with ROCK1 siRNAs. **Supplementary Figure 5.** No significant overexpression of PTGS2 in GSE38129 and GSE29001 databases.

## Data Availability

All experimental data generated or analysed during this study are included in this published article and its supplementary files.

## Abbreviations

AHR: Aryl hydrocarbon receptor
COX2: Cyclooxygenase 2
CYP1A1: Cytochrome P450, family 1, member A1
DIM: 3,3′-Diindolylmethane
EGFR: Epidermal growth factor receptor
ELISA: Enzyme-linked immunosorbent assay
EMT: Epithelial-mesenchymal transition
ESCC: Esophageal quamous cell carcinoma
PGE_2_: Prostaglandin E_2_
qPCR: Quantative Polymerase Chain Reaction
RhoA: Ras homolog gene family member A
ROCK1: Rho-associated coiled-coil kinase
