# Supplementary material for: 3,3′-Diindolylmethane modulates aryl hydrocarbon receptor of esophageal squamous cell carcinoma to reverse epithelial-mesenchymal transition through repressing RhoA/ROCK1-mediated COX2/PGE2 pathway
Source: J Exp Clin Cancer Res. 2020 Jun 16;39:113. doi: 10.1186/s13046-020-01618-7 (PMC7298755; doi:10.1186/s13046-020-01618-7)
Supplement: Supplementary file 1 — Additional file 1 Table S1. Information of antibodies used in WB or co-IP Table S2. Sequences of primer used in qPCR [file 13046_2020_1618_MOESM1_ESM.docx]

**Additional file 1:**

Supplementary tables. Antibodies used in WB or co-IP and sequences of primer used in qPCR

**Table S1.** Information of antibodies used in WB or co-IP

| **Antibody** | **Brand** | **Dilution ratio (WB)** | **Dilution ratio (co-IP)** |
| --- | --- | --- | --- |
| GAPDH | Proteintech, China | 1:5000 | - |
| AHR | Santa Cruz, USA | 1:500 | 1:100 |
| CYP1A1 | Santa Cruz, USA | 1:500 | - |
| β-Catenin | CST, USA | 1:1000 | - |
| Vimentin | Proteintech, China | 1:1000 | - |
| Slug | CST, USA | 1:1000 | - |
| Claudin-1 | CST, USA | 1:1000 | - |
| MMP1 | Proteintech, China | 1:1000 | - |
| MMP2 | OriGene, USA | 1:500 | - |
| RhoA | Abcam, USA | 1:2000 | 1:50 |
| ROCK1 | Abcam, USA | 1:5000 | 1:30 |
| Cofilin | CST, USA | 1:1000 | - |
| p-cofilin | CST, USA | 1:1000 | - |
| F-actin | Abcam, USA | 1:200 | - |
| COX2 | CST, USA | 1:1000 | 1:50 |
| EGFR | Abcam, USA | 1:3000 | 1:50 |
| p-EGFR | CST, USA | 1:1000 | - |
| NF-ΚB p65 | CST, USA | 1:1000 | 1:50 |
| NF-ΚB p-p65 | CST, USA | 1:1000 | - |

**Table S2.** Sequences of primer used in qPCR

| **Gene symbol** | **Sense (5’-3’)** | **Antisense (5’-3’)** |
| --- | --- | --- |
| GAPDH | TCTCTGCTCCTCCTGTTC | GTTGACTCCGACCTTCAC |
| RhoA | ACTGGTGATTGTTGGTGAT | ATCTGCCACATAGTTCTCAA |
| ROCK1 | TTGCTGTGCTGTGAATGA | GGTAATGGTGATGAGTCTGA |
| COX2 (PTGS2) | CGAGGTGTATGTATGAGTGT | AGCCATAGTCAGCATTGTAA |
