# Supplementary material for: 3,3′-Diindolylmethane modulates aryl hydrocarbon receptor of esophageal squamous cell carcinoma to reverse epithelial-mesenchymal transition through repressing RhoA/ROCK1-mediated COX2/PGE2 pathway
Source: J Exp Clin Cancer Res. 2020 Jun 16;39:113. doi: 10.1186/s13046-020-01618-7 (PMC7298755; doi:10.1186/s13046-020-01618-7)
Supplement: Supplementary file 2 — Additional file 2 Supplementary Figure 1. AHR is overexpressed in ESCC and correlates with poor clinical outcomes. A. IHC results showed represented images of AHR expression levels in ESCC. B. AHR was overexpressed in ESCC compared with paired normal tissues. Expression levels of AHR correlated with ESCC clinical stages (C) and lymph node metastasis (D). E. AHR staining-intensities were positively associated with levels of lymph node metastasis. F. GEPIA database indicated overexpression of AHR in ESCA. G. All four ESCC databases downloaded from GEO Dataset verified AHR was overexpressed in ESCC. H. UALCAN database indicated AHR expression levels were significantly associated with tumor histology, tumor grade, lymph nodal metastasis status and clinical stages. * P < 0.05, ** P < 0.01, *** P < 0.001. Supplementary Figure 2. Detection of AHR expression levels in ESCC cell lines by WB. Supplementary Figure 3. Correlation analysis of AHR, RhoA and ROCK1 with GEO Datasets. A: In GSE23400 databases, no significant correlations were found among AHR, RhoA and ROCK1 expression levels. B: In GSE20347, only correlation analysis of AHR and ROCK1 was significant. C: In GSE29001, only correlation analysis of RhoA and ROCK1 was significant. Supplementary Figure 4. Phalloidin staining of F-actin in TE1 (A) and KYSE150 (B) cell lines with ROCK1 siRNAs. Supplementary Figure 5. No significant overexpression of PTGS2 in GSE38129 and GSE29001 databases. [file 13046_2020_1618_MOESM2_ESM.docx]

**Additional file 2**

**
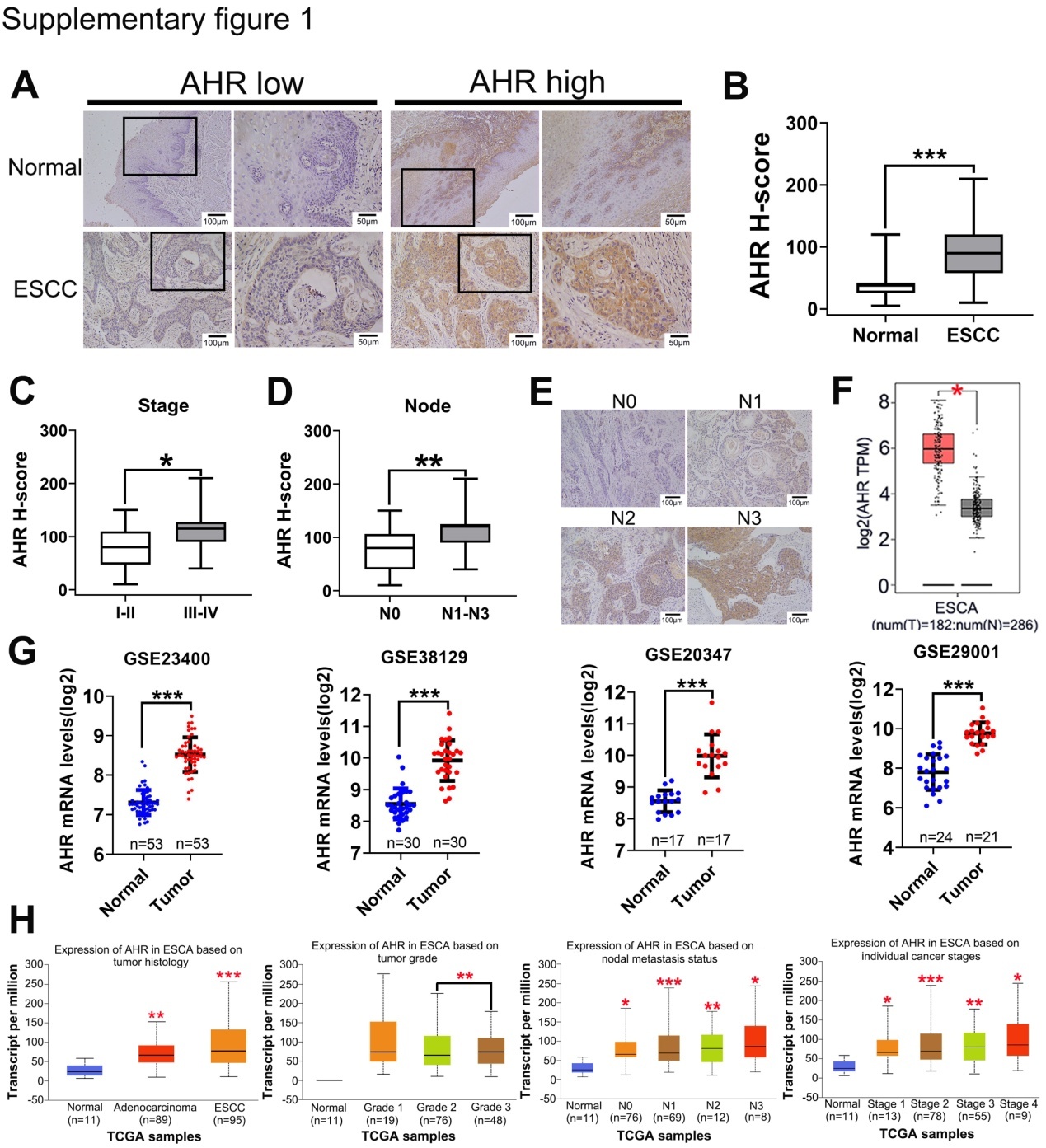
**

**Supplementary figure 1. AHR is overexpressed in ESCC and correlates with poor clinical outcomes.**

A. IHC results showed represented images of AHR expression levels in ESCC. B. AHR was overexpressed in ESCC compared with paired normal tissues. Expression levels of AHR correlated with ESCC clinical stages (C) and lymph node metastasis (D). E. AHR staining-intensities were positively associated with levels of lymph node metastasis. F. GEPIA database indicated overexpression of AHR in ESCA. G. All four ESCC databases downloaded from GEO Dataset verified AHR was overexpressed in ESCC. H. UALCAN database indicated AHR expression levels were significantly associated with tumor histology, tumor grade, lymph nodal metastasis status and clinical stages. * P<0.05, ** P<0.01, *** P<0.001.

**
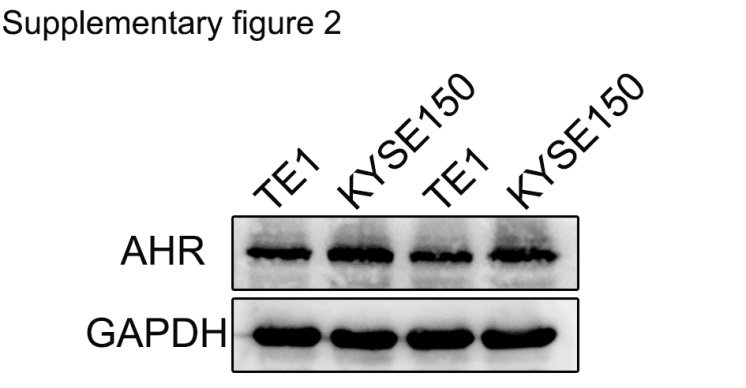
**

**Supplementary figure 2: Detection of AHR expression levels in ESCC cell lines by WB.**

**
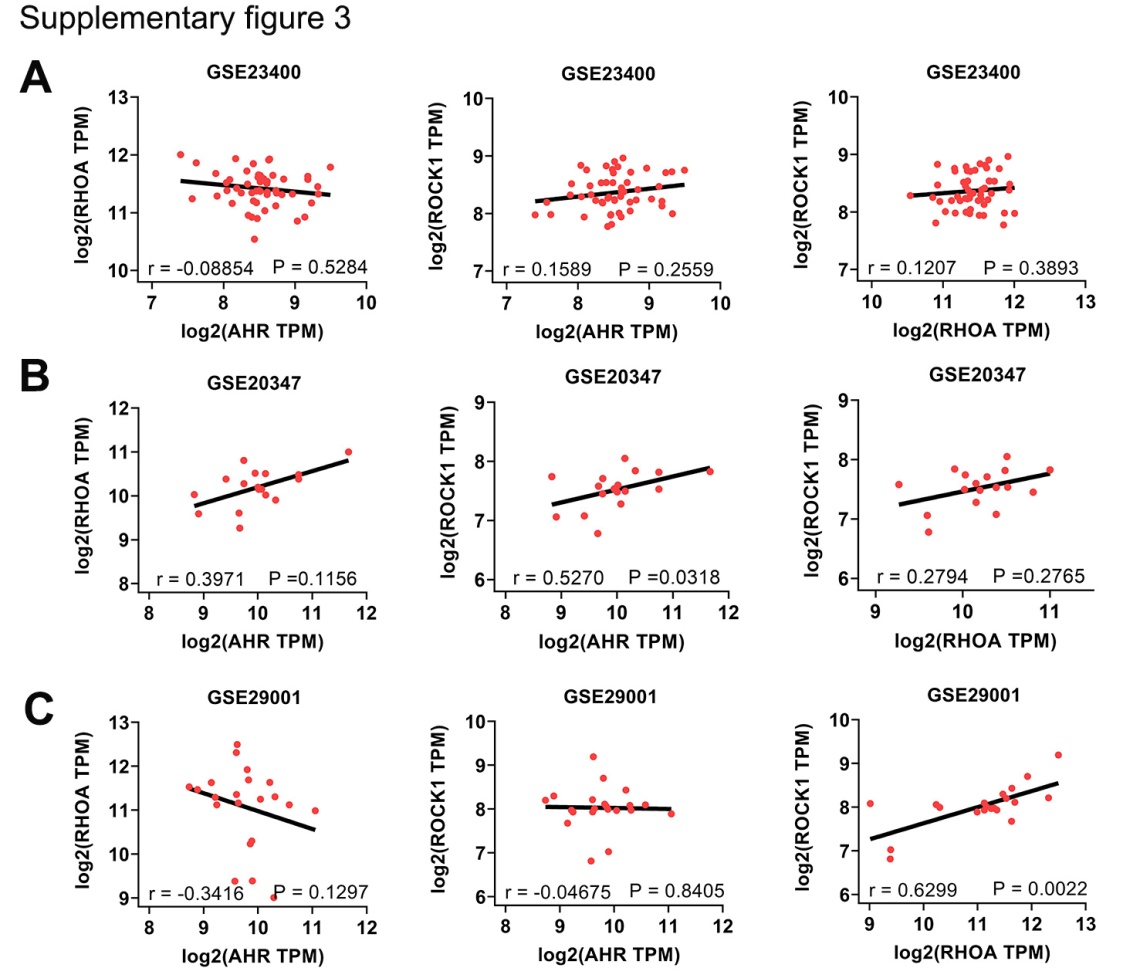
**

**Supplementary figure 3: Correlation analysis of AHR, RhoA and ROCK1 with GEO Datasets.**

A: In GSE23400 databases, no significant correlations were found among AHR, RhoA and ROCK1 expression levels. B: In GSE20347, only correlation analysis of AHR and ROCK1 was significant. C: In GSE29001, only correlation analysis of RhoA and ROCK1 was significant.


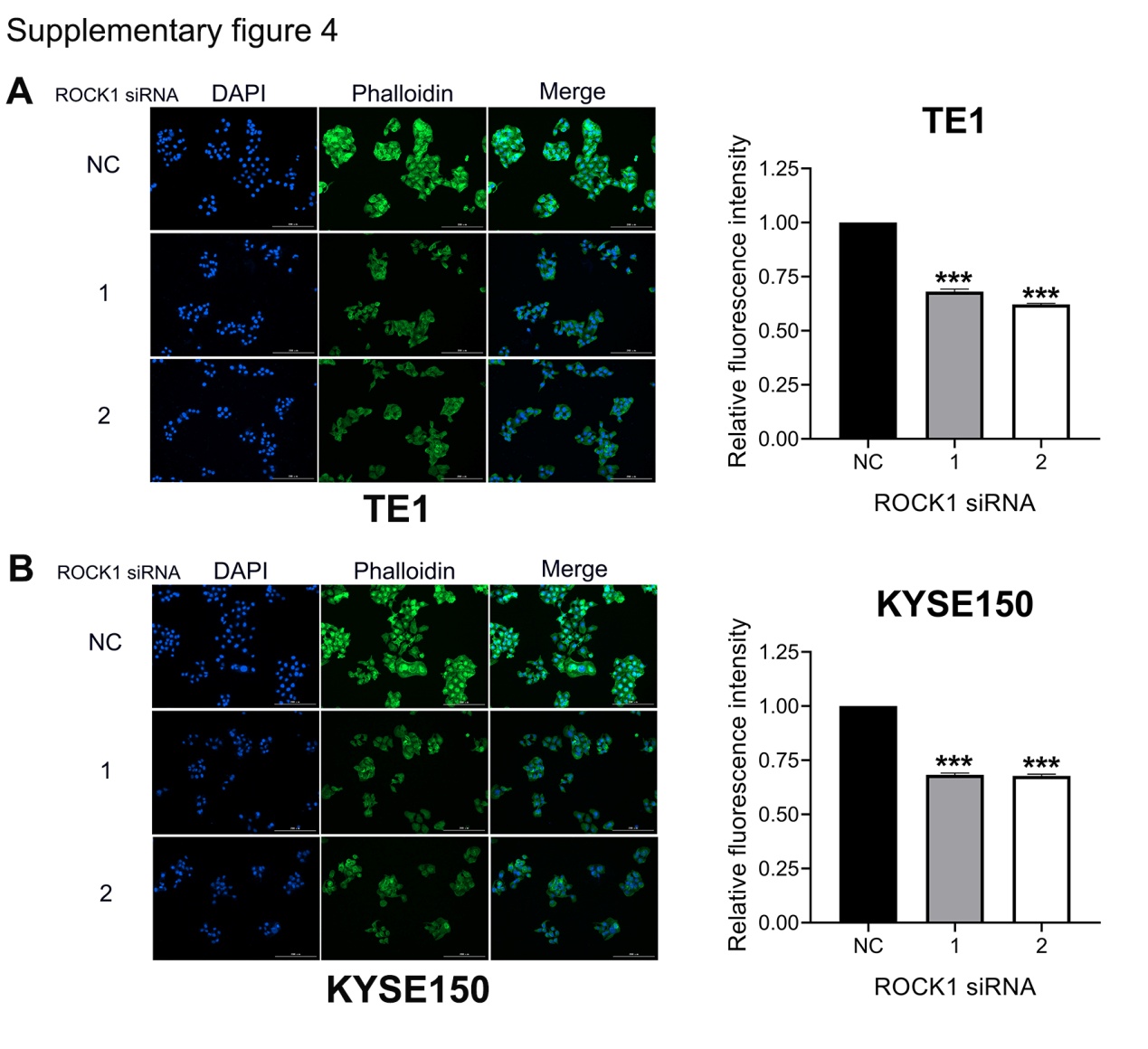


**Supplementary figure 4: Phalloidin staining of F-actin in TE1 (A) and KYSE150 (B) cell lines with ROCK1 siRNAs.**


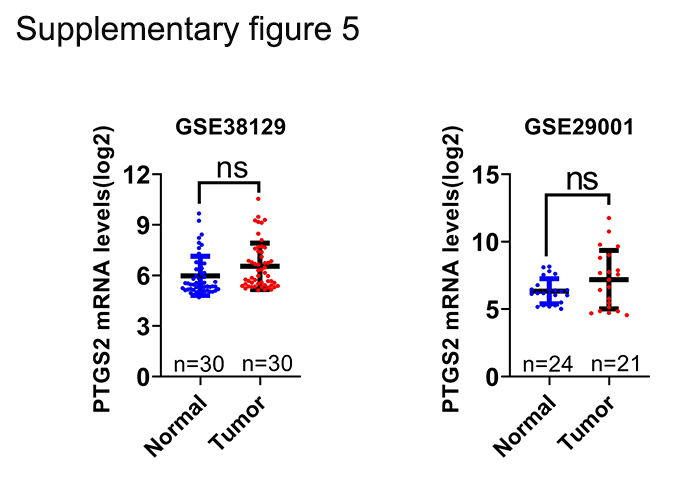


**Supplementary figure 5: No significant overexpression of PTGS2 in GSE38129 and GSE29001 databases.**
